# Holm Oak Somatic Embryogenesis: Current Status and Future Perspectives

**DOI:** 10.3389/fpls.2019.00239

**Published:** 2019-03-22

**Authors:** María Teresa Martínez, María del Carmen San-José, Isabel Arrillaga, Vanesa Cano, Marián Morcillo, María José Cernadas, Elena Corredoira

**Affiliations:** ^1^ Instituto de Investigaciones Agrobiológicas de Galicia (IIAG-CSIC), Santiago de Compostela, Spain; ^2^ ISIC/ERI Biotec/Med, Departamento de Biología Vegetal, Facultad de Farmacia, Universitat de València, Valencia, Spain

**Keywords:** cryopreservation, genetic transformation, oak decline, plant regeneration, *Quercus ilex*, somatic embryos

## Abstract

*Quercus ilex* (holm oak) is one of the most representative trees in the Mediterranean basin, but now the sustainability of its ecosystems is at serious risk due to the lack of natural regeneration and to the presence of a severe disease called oak decline that has caused the death of thousands of trees. The application of biotechnological tools, such as somatic embryogenesis, allows programs of genetic improvement of the species to be speeded up and helps in the conservation of its ecosystems. Somatic embryogenesis is currently considered one of the main biotechnological techniques that has demonstrated significant benefits when has applied to forest tree species, providing significant advantages such as mass propagation, genetic transformation, application of synthetic seed, and cryopreservation of elite genotypes. In this report, the state of the art of somatic embryogenesis in holm oak is reviewed. Factors affecting the induction (plant donor age, type of explant, or plant growth regulators) and maintenance and proliferation of the embryogenic cultures are summarized. Advances on the conversion of somatic embryos into plants and on the acclimatization of these plantlets, as well as the results obtained on the application of the genetic transformation and the cryopreservation procedures to holm oak embryogenic cultures, are also presented.

## Introduction

The genus *Quercus* is one of the most important clades of woody angiosperms in the northern hemisphere (Nixon, 2006) and includes both deciduous and evergreen species. In the Mediterranean basin, holm oak (*Quercus ilex* L.*),* a sclerophyllous evergreen tree, is a structural species, which is distributed throughout the Iberian Peninsula, the south east of France, Morocco, Algeria, and Italy (Villar-Salvador et al., 2013). In the Iberian Peninsula, populations of holm oak, in most cases, grow together with other trees, in particular *Pinus* species (Rodà et al., 2009) and other *Quercus* species (i.e., *Q. suber, Q. coccifera*) (Díaz and Pulido, 2009). All these species are distributed in the wild and in managed forests, where they display a remarkable capacity to adapt to local conditions and where holm oak is the dominant tree species (González-Rodríguez et al., 2011). In managed forests, holm oaks are located in the artificially created and maintained oak woodland systems, known locally as *dehesas* (in Spanish) or *montados* (in Portuguese), subjected to a strong human intervention throughout the centuries from the original Mediterranean forest (Smit et al., 2009; Huntsinger et al., 2013), and now the semi-natural forests are limited to small areas of difficult access (Blanco et al., 2005). The conservation of the holm oak woodlands and *dehesas* is a key requirement for the perpetuation of the Mediterranean forest biological diversity. For this reason, *dehesas* and holm oak woodlands were included in the Special Areas of Conservation defined in EU Council Directive 92/43/EEC (Council of Europe, UNEP and ECNC, 1996). On the order hand, the Declaration of the *Dehesas de Sierra Morena* as Biosphere Reserve by UNESCO in 2002 involves the recognition and support of this system of exploitation. *Dehesas* constitute an agrosilvopastoral system of great economic importance for rural areas in which vegetation (oak woodland, croplands, grasslands, and shrub lands) is commonly used for the breeding of livestock in an extensive system. *Dehesas* produce a variety of highly valued products, including Iberian ham, one of the most valuable cured hams in the world (Vargas et al., 2013); black truffle, one the most expensive edible fungi in international *haute cuisine* (Reyna-Domenech and García-Barreda, 2009); merino wool, cheese, and sheep and goat meat (Vargas et al., 2013); and firewood harvesting, beekeeping, and hunting (Moreno and Pulido, 2009; Huntsinger et al., 2013). Recently, a pop-up activity has been introduced, tourism (Pulido et al., 2013).

In the last few years, *dehesas* are facing an unprecedented crisis due to the high mortality of evergreen oak trees, which has progressively increased throughout southern Europe and North Africa (Linaldeddu et al., 2014). Holm oak decline, also called *“la seca”* (in Spanish) or *“a secca”* (in Portuguese), has a multiple etiology characterized by non-specific symptoms: treetop thinning, wilting of leaves, twigs and branches, twig dieback, exudations from bark and root lesions, and proliferate epicormic shoots (Natalini et al., 2016). It is a complex syndrome caused by biotic and abiotic factors. Among the biotic factors involved in this syndrome, a primary role is played by several fungal pathogens such as *Biscogniauxia mediterranea*, *Diplodia corticola*, *Discula quercina*, and mainly by the oomycete *Phytophthora cinnamomi* (Maddau et al., 2011; Linaldeddu et al., 2014). The root pathogen, *Phytophthora*, acts progressively, destroying the system of fine roots of affected holm oak trees, making them more susceptible to droughts and attacks by other pests, and causing the decline and a rapid mortality of trees (Corcobado et al., 2013; Pérez-Sierra et al., 2013; Jung et al., 2016). However, its decline has occurred not only due to pathogens but also due to abiotic factors as a result of the abandonment of traditional uses (Plieninger et al., 2004), low regeneration capacity of the trees (Pulido et al., 2013), and episodic events of drought (Corcobado et al., 2014).

## Why Somatic Embryogenesis on Holm Oak?

Due to the great complexity of the oak decline syndrome, the solutions to be applied to restore the affected ecosystems are not easy. To our knowledge, conventional breeding programs, including large backcross steps, have not been, and probably will not be, carried out in holm oak to obtain disease tolerant/resistant plants, as were carried out in both, the European chestnut (Vieitez et al., 1986) and the American chestnut (Anagnostakis, 2001). The most common recommendation for prevention against oak decline is the creation of new and healthy stands. Firstly, it is necessary to facilitate and improve natural regeneration of the stands, but artificial reforestation with the aim of a progressive substitution of decrepit trees for young seedlings in a stand also prevents against the spreading of the disease towards other trees or nearby stands (Montoya and Mesón, 2004). Several studies have described genetic differences in holm oak populations in relation to the susceptibility/tolerance to infection by *P. cinnamomi* (Navarro et al., 2004; Tapias et al., 2006). The capacity of some individuals to survive in areas strongly affected by oak decline and the results of greenhouse trials in which *Q. ilex* was artificially infected with *P. cinnamomi* indicate a high degree of genetic variation of oomycete tolerance in the species (Tapias et al., 2006). The vegetative propagation of these tolerant trees could be a possible alternative to producing improved plant stock (clonal forestry). However, holm oak is considered recalcitrant to vegetative propagation, showing poor rooting ability of cuttings, which is also reduced with the aging of parent plants (L’Helgoual’ch and Espagnac, 1987). Different reviews have described the possibilities of forest biotechnology as an emerging opportunity in relation to tree improvement (Merkle and Nairn, 2005; Pijut et al., 2011; Vieitez et al., 2012). Biotechnological tools, and specifically, micropropagation techniques, can help overcome the above mentioned problems, providing methods for large-scale propagation and germplasm conservation of tolerant and/or resistant holm oak trees. Although standardized procedures for the micropropagation by axillary shoot proliferation of the most important oak species have been reported (Vieitez et al., 2012), in holm oak, there are only two reports that describe its propagation by this way. Liñan et al. (2011) reported the regeneration of this species through axillary shoot proliferation from juvenile material. Recently, shoots obtained after forced sprouting of crown branch segments collected from 30- and 100-year-old trees were used to establish axillary shoot cultures (Martínez et al., 2017a). Besides the reduced number of reports on axillary budding, these protocols still need to be optimized, not only for shoot proliferation but also for shoot rooting and acclimatization.

Conversely, more positive results have been reported in holm oak propagation by somatic embryogenesis. This micropropagation *via* is considered the most appropriate method for clonal propagation of woody species due to its high multiplication potential (Lelu-Walter et al., 2013; Guan et al., 2016). Somatic embryogenesis allows a large number of genetically identical individuals to be obtained that can be used in improvement programs and to establish germplasm banks. Somatic embryogenesis has also been revealed as the best way of regeneration in cryopreservation (Engelmann, 2004) and genetic transformation procedures (Giri et al., 2004), and it is increasingly important in functional genomics studies, in order to validate genes related to the embryogenic process. The application of bioreactors with continuous or temporal immersion systems (TIS) for culture of somatic embryos helps to improve the quality of the embryos, to increase the proliferation and conversion rates, and at the same time to reduce the production costs (Fei and Weathers, 2015). Somatic embryogenesis also facilitates the production of synthetic seeds by applying encapsulation techniques to the somatic embryos (Guan et al., 2016). Finally, somatic embryogenesis is considered an effective strategy to speed up the deployment of outstanding families identified in progeny trials when introduced into multi-varietal forestry tree breeding programs (for a review see Park et al., 2006, 2016). Hence, somatic embryogenesis can be considered an efficient alternative for the large-scale production of holm oak trees.

This report reviews the achievements and current status of somatic embryogenesis induction in oak species with special reference at holm oak. The main results obtained on the application of somatic embryos to define cryopreservation and genetic transformation procedures in holm oak are also summarized.

## Advances on the Application of Somatic Embryogenesis in Adult Trees: Oak Species as an Example

The genetic improvement of forest species is a slow process due to the common tree biology: slow growth, late flowering, and high degree of heterozygosis that limits the use of conventional improvement techniques. On the other hand, the characteristics of commercial interest, such as fruit production, quantity and composition of wood, and resistance/tolerance to biotic and abiotic stresses, are usually of a polygenic nature or are fixed to recessive alleles that make difficult for their selection with classical breeding techniques. In this sense, vegetative propagation, specifically somatic embryogenesis, is important for the production of selected genotypes, as well as reducing the selection cycles in genetic breeding programs (Niemi et al., 2004).

Nowadays, the main limitation for using somatic embryogenesis in trees is that, in many Hardwood species and in almost all Gymnosperm species, the induction of somatic embryos is achieved by using zygotic embryos as an initial explant, which normally have a genetic unknown value (Corredoira et al., 2018a). It would be recommendable to induce somatic embryogenesis in explants from adult specimens when the characteristics of the genotype that is being propagated are known; but then, the capacity to induce somatic embryogenesis decreases considerably (Bonga et al., 2010). This is due to the fact that woody species undergo a phase change phenomena or ontogenetic aging during their development. This process is defined as progressive transition from the juvenile state to the adult state, which is characterized by a reduction in growth, start of flowering, and a significant decrease in organogenic and embryogenic capacity (von Aderkas and Bonga, 2000; Díaz-Sala, 2016).

A possible way to overcome this problem is to employ adult tissues that have regained juvenile characteristics like floral tissues (e.g., immature inflorescences, petals, floral staminodes, pistils, stamens, or anther teguments) or maternal tissues (e.g., nucellus or inner teguments) (Corredoira et al., 2018a). The induction of somatic embryos is generally easier in these types of explants, as the site of meiosis is closed around the time of meiosis (Bonga et al., 2010). The use of these tissues has allowed the induction of somatic embryos from adult trees in species such as *Aesculus hippocastanum* (Jörgensen, 1989), *Hevea brasiliensis* (Carron et al., 1995), *Liriodendron tulipifera* (Merkle and Battle, 2000), or *Theobroma cacao* (Li et al., 1998).

Leaves from adult trees can also be used as explants for inducing somatic embryogenesis and are more abundant and easier to manage than floral tissues. The origin of the leaves used as the initial explant for inducing somatic embryos is of key importance. Although there are examples in which somatic embryogenesis is initiated from leaves collected directly from the adult tree such as coffee (Etienne, 2005), these explants however seem not to be adequate in recalcitrant species. In the last few years, extensive work has been performed in oak species, and as a result of this, several solid and successful procedures for induction of somatic embryogenesis from adult trees have been defined (Corredoira et al., 2014). Initially, the objectives were focused on the search within the tree material that had undergone a certain level of rejuvenation. First, a successful protocol for inducing somatic embryos in recalcitrant cases was defined using leaves obtained from shoots derived from forced flushing of branch segments (Ballester et al., 2016). Forced shoots are the result of growth of preformed dormant buds, which are in a state of quiescence in the tree (Vieitez et al., 2012). These shoots usually grow rapidly and have long internodes and more juvenile looking leaves. These expanding leaves have been used as initial explants for somatic embryogenesis induction in mature trees of *Q. suber* (Hernández et al., 2003a) and *Q. robur* (Toribio et al., 2004; Corredoira et al., 2006).

**Figure 1:**
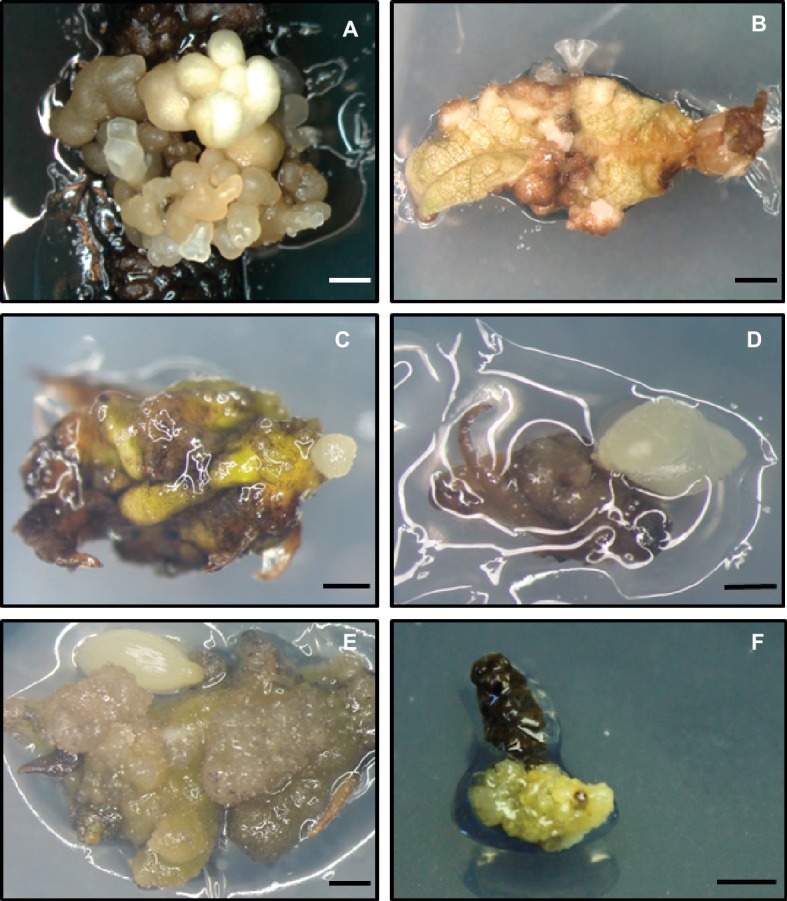
Somatic embryogenesis induction on different explants derived from adult trees of *Quercus* spp. **(A,B)** Somatic embryos formed on leaf explants excised from *in vitro* shoot cultures of *Q. robur* **(A)** and *Q. alba* **(B)**. **(C)** Globular-stage somatic embryo generated on shoot tip of *Q. rubra*. **(D,E)** Somatic embryos induced on apex **(D)** and leaf **(E)** explants excised from axillary shoot cultures of *Q. ilex*. **(F)** Embryogenic callus generated from a male catkin of *Q. ilex*. Scale bar: 1 mm.

A recent highly recommended alternative for somatic embryogenesis induction in adult trees is the use of *in vitro* shoot cultures as an initial explant source (Corredoira et al., 2018a). Axillary shoot cultures produce uniform explants and guarantee year-round unlimited supply, as flushing of branch segments is not required after *in vitro* establishment of the donor shoots (Ballester et al., 2016). This material serves to provide not only leaves but also other types of initial explants, such as shoot tips, nodes, and internode segments. Additionally, the successive subcultures in a culture medium with cytokinins produce some rejuvenation in the shoots, which could improve the embryogenic capacity of these cultures (Wendling et al., 2014). Axillary shoot cultures have also been successfully used to induce somatic embryos in apex and leaf explants of *Q. robur* (San José et al., 2010), *Q. alba* (Corredoira et al., 2012), *Q. bicolor* (Mallón et al., 2013), and *Q. rubra* (Martínez et al., 2015) (Figures 1A–C). In all of them, higher embryogenesis induction rates were obtained in leaf compared to apex explants. Moreover, explants derived from *in vitro* shoot cultures have also been used to induce somatic embryogenesis in other important woody species such as elm (Conde et al., 2004), tamarind (Correia et al., 2011), eucalyptus (Corredoira et al., 2015), or strawberry (Martins et al., 2016).

Another factor to take into account is the position of the explant on the shoot, with the chronologically younger ones being the most distal. In general, for somatic embryogenesis induction, the parts of the plants with active growth and near to the apices such as semi-expanded apical leaves or the most apical internodes/nodes are usually used. In oak species, when leaves were excised from forced flushing of branch segments, as well as from axillary shoots *in vitro* cultures, it is imperative to culture the first or second leaf from the apex. For example, in *Quercus alba*, the embryogenic response was inversely proportional to leaf position in the shoot, as the two younger leaves showed a higher embryogenesis percentage (51%) than the third and fourth leaves (19 and 4%, respectively) (Corredoira et al., 2012). Anatomical study of these leaves showed that the first leaf has less degree of differentiation (with the presence of precursor guard cells of stomata, absence of intercellular spaces, and low starch content in the mesophyll) than the fourth one (Corredoira et al., 2014). In *Q. suber,* the most appropriate physiological state of the leaves was determined by their size, as only leaves of 1.5 cm or less showed successful response to the induction process (Hernández et al., 2003a). Therefore, recently formed, that is to say, very young and scarcely differentiated leaves, should be used.

Besides the influence of explant type and its physiological stage, another important question on somatic embryogenesis procedures is the composition of induction medium. Among the all components of induction medium, it is well established that the auxins and cytokinins are the most important compounds (Fehér, 2015). Naphthalene acetic acid (NAA) and 2,4-dichlorophenoxyacetic acid (2,4-D) either alone or in combination with a cytokinin are the auxins mostly used to induce somatic embryos in leaves of Hardwood species (Corredoira et al., 2018a). Apical shoot apices and the most apical expanding leaf with petiole from the first node in the apical region of pedunculated oak (Toribio et al., 2004; San José et al., 2010), cork oak (Hernández et al., 2003a), white oak (Corredoira et al., 2012), swamp oak (Mallón et al., 2013), and red oak (Martínez et al., 2015) were cultured following a three-step method. This procedure involves successive culture of explants in three media: (1) induction medium (M1) consisting of Murashige and Skoog basal medium (Murashige and Skoog, 1962) supplemented with high concentrations of NAA (10 mg/L in the case of cork oak and 4 mg/L in the rest of oak species) in combination 6-benzyladenine (BA) for 8 weeks; (2) M2 medium consisting of induction medium but with auxin and cytokinin reduced to 0.1 mg/L for 4 weeks; and (3) expression medium (M3) consisting of induction medium without PGRs for at least 12 weeks.

## Holm Oak Somatic Embryogenesis: Where Do We Stand at Present?

### Initiation of Embryogenic Cultures

In the last few years, several protocols for propagation of holm oak by somatic embryogenesis have been defined in spite of this species is considered a very recalcitrant species. Somatic embryos have been initiated from different types of initial explants including zygotic embryos, male catkins, ovule teguments, leaves, and shoot apices.

#### Initiation From Zygotic Embryos

When zygotic embryos are used as initial explants, their development stage is the most limiting factor for somatic embryogenesis to be possible (Corredoira et al., 2018a). Zygotic embryos usually show embryogenic capacity in a determined stage of its ontogenetic development called “developmental window” (Trigiano et al., 1999). This stage corresponds with a short period of time prior to maturation, in which the induction of somatic embryos is easier (Merkle et al., 1995). In holm oak, somatic embryogenesis was obtained only with zygotic embryos collected in August, while no positive results were obtained in zygotic embryos collected in July or September (Mauri and Manzanera, 2003). Zygotic embryos were cultured on Gamborg medium (Gamborg et al., 1968) supplemented with 1.9 mg/L NAA and 2.2 mg/L BA during a month. Subsequently, explants were cultured on G medium with 1.1 mg/L BA and 0.95 mg/L NAA for 30 days and finally transferred to basal medium without plant growth regulators (PGRs) for 30 days. Initial response was the formation of a white-translucent callus, which generated embryo-like structures 3–8 weeks after the start of the experiments (Mauri and Manzanera, 2005). Commonly, immature zygotic embryos have a greater embryogenic capacity than other explants used for somatic embryogenesis induction (Merkle et al., 1995; Guan et al., 2016). However, in holm oak, the embryogenic response obtained from zygotic embryos is rather low (4.3%) (Mauri and Manzanera, 2005), particularly if it is compared with that obtained in other oak species where relatively high induction rates (> 70%) have been attained (Corredoira et al., 2014). These low induction rates are probably due to the use of the zygotic embryos not in an optimal stage of development, which is generally defined when it is in the globular to early cotyledonary stage (Corredoira et al., 2018a).

#### Initiation From Mature Explants

Holm oak can be an example of what is possible in the induction of somatic embryos from adult material, if a careful choice of the initial explant and of its development stage is made. In early reports, the induction of somatic embryogenesis in leaves directly collected from mature trees of holm oak (50 years old) was described, but somatic embryos were not able to regenerate plantlets (Féraud-Keller et al., 1989; Féraud-Keller and Espagnac, 1989). Leaves were cultured on MS with 4 mg/L BA and 0.5 mg/L NAA. Secondary nodules that later produce somatic embryos in a percentage of 3% were only observed on leaf explants collected on October. The results of that study could not be repeated when expanding leaves from sprouted epicormic shoots were used (Blasco et al., 2013; Barra-Jiménez et al., 2014). Recently, somatic embryogenesis has been achieved from leaf and apex explants excised from axillary shoot proliferation cultures established from two mature *Q. ilex* trees (Martínez et al., 2017b). Explants were cultured following the three-step procedure previously described for other oaks, but in holm oak two M1 media were evaluated, one supplemented with 4 mg/L NAA plus 0.5 mg/L BA and another with 4 mg/L IAA plus 0.5 mg/L BA. The best results were obtained with apex explants (11%) cultured on NAA (Figure 1D), although without significant differences with the treatment including IAA. The treatment with 4 mg/L NAA and 0.5 mg/L BA was also effective for initiating somatic embryogenesis from leaf explants (Figure 1E), but induction frequencies were lower (between 1 and 3%, depending on the genotype). It should be noted that holm oak somatic embryogenesis has also been obtained at low frequency on leaf and shoot apex explants cultured on PGR-free medium, which indicates that both explant types in an appropriate developmental stage have a significant embryogenic capacity. Unlike that described for other species of the *Quercus* genus (Corredoira et al., 2014; Martínez et al., 2015), the apices showed a greater embryogenic response than the leaves. This is mainly because holm oak leaves tend to necrose and end up dying during the culture on M1 medium. Moreover, they have a higher degree of differentiation than that observed in leaves for other oak species (Corredoira et al., 2006, 2012). By contrast, the leaf primordia attached to the axillary bud of apex explants (less differentiated meristems) are able to survive and seem to be the source of initial calli, and subsequently of the embryogenic response (Martínez et al., 2017b). Besides, histological studies showed that the number of leaf primordia attached to the apex is higher in holm oak than in other oak species. This fact can be a possible explanation for the greater embryogenic capacity of the apex in holm oak. It is important to highlight that the percentages obtained in shoot tips for holm oak are superior to those mentioned for *Quercus bicolor* (Mallón et al., 2013) and *Quercus rubra* (Martínez et al., 2015), being 6.9% and 2.3%, respectively. At the same time, induction rates on holm oak are similar or higher than those mentioned for *Quercus robur* where, depending of the genotype, percentages of 1.4%–11.8% have been reported (San José et al., 2010).

Embryogenic cultures from holm oak adult trees have also been achieved from floral tissues. Blasco et al. (2013) induced somatic embryos in one out of five tested genotypes (frequency ranging from 0.2 to 2.3%) when both isolated male flowers and catkins were cultured following a three-step method on M1 medium with 2 mg/L or 10 mg/L NAA plus 2.2 mg/L BA (Figure 1F). Barra-Jiménez et al. (2014) obtained embryogenic lines from two of four tested genotypes from teguments of developing ovules. The highest induction frequencies (frequency ranging from 1.2 to 3.2%) were achieved when ovules excised at an advanced stage of development (i.e. at least 3–4 mm wide and the rest of the ovules within the ovary had aborted) were cultured on medium Schenk and Hildebrandt (1972) without PGRs. In both explant types, induction rates were lower than that obtained from apices, although in all explants used the genotype of donor tree has great influence on the induction rates (Blasco et al., 2013; Barra-Jiménez et al., 2014; Martínez et al., 2017b). With exception of somatic embryogenesis induction on teguments, holm oak somatic embryos initiated in other explant types have been obtained using a three-step procedure in which the initial high auxin concentration used was reduced at first and finally removed. In the majority of studies, the somatic embryos are generated indirectly after to callus formation (Féraud-Keller and Espagnac, 1989; Mauri and Manzanera, 2005; Blasco et al., 2013; Barra-Jiménez et al., 2014; Martínez et al., 2017b), although in the case of teguments, direct formation of single embryos was occasionally observed (Barra-Jiménez et al., 2014).

### Somatic Embryo Maintenance

The maintenance of embryogenic ability is performed using: (1) secondary or repetitive embryogenesis from isolated somatic embryos in torpedo-early cotyledonary stages which formed secondary embryos and, (2) culture of nodular embryogenic structures (NSs) or pro-embryogenic masses (PEMs) (Merkle et al., 1995; Corredoira et al., 2018a). Usually, in the majority of Hardwood species, proliferation of somatic embryos was not a critical step. Indeed, somatic embryos or/and embryogenic callus generated on the initial explants were isolated and cultured in proliferation medium to produce numerous somatic embryos by secondary embryogenesis. However, the maintenance of embryogenic ability in holm oak is one the main bottlenecks as most of the initial embryogenic cultures lost their embryogenic competence after few subcultures (Blasco et al., 2013; Barra-Jiménez et al., 2014; Martínez et al., 2017b; Corredoira et al., 2018b). Furthermore, after isolation, some primary embryos underwent rapid differentiation to the cotyledonary stage, sometimes even forming complete plants without developing secondary somatic embryos. Another limitation is that in holm oak embryogenic cultures generated on initial explants are usually formed by one creamy translucent nodular structure or one somatic embryo at a different developmental stage, which is a handicap to the subsequent establishment of the different embryogenic lines. By contrast in other oak species, initial embryogenic cultures are able to proliferate by secondary embryogenesis in the initial explant, and then, the establishment of embryonic lines is easier (Corredoira et al., 2014). In holm oak, the most efficient method defined up until now in order to establish embryogenic lines requires isolating the somatic embryos and/or NSs in very early stages of their development before starting their histodifferentiation (Martínez et al., 2017b).

**Figure 2:**
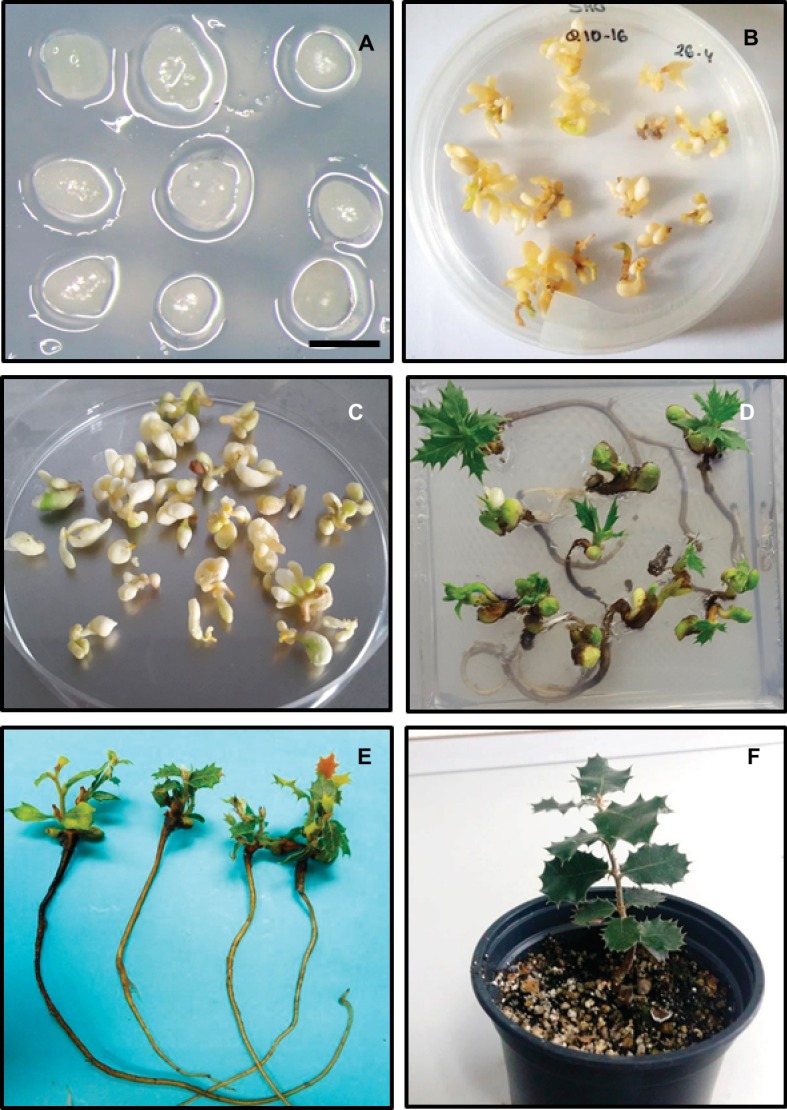
Somatic embryo proliferation and plant regeneration on holm oak. **(A)** Morphological aspect of nodular embryogenic structures used as inocula explants in proliferation, cryopreservation, and genetic transformation experiments. Scale bar: 1 mm. **(B)** Somatic embryos originated from nodular embryogenic structures showed in A after 6 weeks of culture on proliferation medium. **(C)** Morphological aspect of 2-month chilled mature somatic embryos. **(D,E)** Plantlets regenerated from somatic embryos subjected to cold treatment for 2 months and 6 weeks in germination medium. **(F)** Plantlet following two seasons of growth in the greenhouse.

Irrespective of their juvenile or adult origin, all holm oak embryogenic cultures were maintained on SH medium without PGRs (Mauri and Manzanera, 2005; Blasco et al., 2013; Barra-Jiménez et al., 2014; Martínez et al., 2017b). Proliferation rates were frequently low in spite of the different culture conditions and/or media tested (Blasco et al., 2013; Barra-Jiménez et al., 2014). To improve proliferation rates, developmental stage of the explant inocula used as initial explant during embryo proliferation was evaluated by Martínez et al. (2017b). Nodular embryogenic structures or PEMs (Figure 2A) were the most effective explant and produced the highest significant numbers of PEMs and secondary embryos (Figure 2B). Low embryo multiplication rates were obtained when torpedo or early cotyledonary-stage somatic embryos were used as the initial explant for embryo proliferation. Similarly, Martínez et al. (2015) also showed that NSs were the most effective explant to embryo production and differentiation of new secondary embryos in American oak species, despite being the smallest explant type in terms of size and fresh weight. These results pointed out the importance of developmental stage of the embryogenic explant used for subculture on somatic embryo proliferation ability.

The addition of organic nitrogen form has been mentioned as important factor for embryo proliferation (Merkle et al., 1995). Usually amino acids, such as glutamine, proline, or alanine, and/or amino acid complexes, such as casein hydrolysate or malt extract, are added to embryo proliferation medium. Among them, glutamine and casein hydrolysate, supplemented separately or all together, are the most used (Corredoira et al., 2018a). However, they were generally not systematically compared and it is no clear whether one is superior to another or even if either is necessary. Concerning holm oak, the addition of glutamine or casein hydrolysate to proliferation medium had a detrimental effect on embryo multiplication (Martínez et al., 2017b).

Embryo culture on liquid medium (suspension culture or TIS) has also been evaluated to enhance somatic embryo production and the synchronization of embryo development culture of somatic embryos. Mauri and Manzanera (2003) observed that culture in liquid medium provided a significantly higher fresh weight of somatic embryos and a higher rate of secondary embryogenesis than semisolid medium. By contrast, Barra-Jiménez (2015) obtained an increase on the proliferation of some of the tested embryogenic lines when TIS was applied, but it did not promote the differentiation of single cotyledonary embryos.

### Maturation and Plantlet Conversion

Several problems have been pointed out with respect to plantlet production from somatic embryos in woody plants. Usually, germination with root-only development is not problematic, whereas the percentage of somatic embryos with simultaneous development of shoot and root (plantlet conversion) is low (Ballester et al., 2016). Another important problem is related to the quality of regenerated plantlets, which normally show less vigor, deficient root development, and lower length than those derived from zygotic embryo germination.

These problems mainly arise from a defective maturation and/or poor apical shoot development (Corredoira et al., 2018a). The usual procedure for the maturation of somatic embryos is their culture in media with high osmolarity or with abscisic acid (ABA), which induces the synthesis of storage products and a decrease in the water content (von Arnold, 2008). One way to restrict water uptake is to culture somatic embryos with permeating osmotic (e.g., high concentration of sugars or sugar-alcohols) or non-permeating osmotic (e.g., polyethylene glycol (PEG) or dextran) agents. In holm oak, somatic embryo maturation with osmotic agents has scarcely used. Mauri and Manzanera (2004, 2005) observed that sucrose concentration significantly influenced germination, and the best germination rates were obtained between 3 and 15% sucrose.

In seeds, the role of ABA during maturation is associated with the inhibition of precocious germination, the synthesis of the reserve substances, acquisition of tolerance to desiccation, and the induction of dormancy (Kermode, 1995). Although ABA and PEG are extensively applied on the maturation of conifer somatic embryos (Stasolla et al., 2002; von Arnold, 2008), there are many inconclusive results in hardwood species. Holm oak is the only example among the *Quercus* genus, in which the presence of ABA (0.1 or 1 μM) on maturation media has a positive effect on plant regeneration, reducing significantly unwanted secondary embryogenesis (Mauri and Manzanera, 2004).

In many woody species, the transfer of the somatic embryos directly for the maturation medium to the germination medium leads to poor plantlet conversion and/or an abnormal development of plantlets. This makes it necessary to apply a series of treatments grouped under the name of pre-germination treatments, prior to the germination and plant development. The purpose of these treatments is to break the dormancy imposed by the ABA or by the osmotic agents, to increase the levels of gibberellic acid, to stimulate germination, and to synchronize the simultaneous development of the root and the shoot (Merkle et al., 1995; Gaj, 2004). The main pre-germination treatments are desiccation, cold storage, and the addition of gibberellic acid to the germination medium (Merkle et al., 1995). In holm oak, the best results for plantlet conversion have been obtained after application of 2 months of cold storage with (Mauri and Manzanera, 2004) or without a previous maturation treatment (Barra-Jiménez et al., 2014; Martínez et al., 2017b). This pre-germination treatment has also stimulated somatic embryo germination and plantlet conversion in cork oak (Fernández-Guijarro et al., 1995; Hernández et al., 2003b), as well as chestnut species (Corredoira et al., 2003, 2008; Andrade and Merkle, 2005). Holm oak somatic embryos are stored at 4°C on SH medium (Mauri and Manzanera, 2005; Barra-Jiménez et al., 2014) or empty Petri dishes, which also induce simultaneously a starvation treatment and partial desiccation during cold storage (Martínez et al., 2017b) (Figure 2C). Cold treatment is highly recommended for somatic embryo maturation in the case of species in which the seeds experience a process of cold stratification in nature, as is the case of oak species (Corredoira et al., 2018a).

Somatic embryo germination has been attained on SH medium without PGRs (Mauri and Manzanera, 2004; Barra-Jiménez et al., 2014), but the addition of 0.05 mg/L BA plus 0.05 mg/L IBA to the germination medium improved the frequency of plantlet recovery up to 36% from 25% in PGR-free medium (Barra-Jiménez et al., 2014). Recently, acceptable conversion rates (21–50%) were achieved when stratified somatic embryos were cultured on Gresshoff and Doy medium (Gresshoff and Doy, 1972) supplemented with 0.1 mg/L BA and 20 μM silver thiosulfate (Martínez et al., 2017b).

Plant regeneration in holm oak is genotypic dependent as it has been mentioned in other hardwood species (e.g. Sánchez et al., 2003; Hernández et al., 2003b; Andrade and Merkle, 2005; Corredoira et al., 2008). Nevertheless, the genotype effect is less in holm oak than in other oak species studied and plantlet conversion rates have improved after optimization of proliferation and maintenance of embryogenic lines (Corredoira et al., 2018b). In addition, good quality of plants in terms of shoot length and leaf development is observed in holm oak (Figures 2D,E). The genotype, the correct formation of the caulinar apex, and an adequate accumulation of storage reserves during maturation step are considered as main factors to achieve a successful plant regeneration (Merkle et al., 1995; Corredoira et al., 2018a).

### Plantlet Acclimatization

Practical application of somatic embryogenesis to propagate selected trees is only considered attained when the successful acclimatization of a large number of plants to field conditions is produced (Pinto et al., 2013). Regarding the acclimatization step on holm oak, the available information is still very limited. Barra-Jiménez et al. (2014) not observed survival on somatic plantlet transferred to forest containers filled with a mixture of peat and perlite. Later on, Martínez et al. (2017b) achieved the acclimatization of somatic plantlets and potted them in moistened terrahum:perlite (1:2) (Figure 2F), but the survival rates were low (less than 10%).

## Cryopreservation of Embryogenic Cultures

The application of cryopreservation techniques or storage in liquid nitrogen (−196°C; LN) to plant cells enables them to be stored for long periods with minimal maintenance, cost, and risks of somaclonal variation (Sakai, 1995). Cryopreservation offers the possibility of creating germplasm banks that would not only help in the storage of plant materials selected for their characteristics but also for the conservation of threatened plant species or species with recalcitrant seed that cannot be stored for long periods of time, as in the case of the holm oak (Engelmann, 2011). To date, cryopreservation has been applied in woody plants, for the conservation of callus cultures, dormant buds, apical meristems, embryonic axes, seeds, embryogenic cultures, and pollen (Engelmann, 2011; Corredoira et al., 2017a). Among these, somatic embryos are considered an ideal explant for cryopreservation procedures (Engelmann, 2011). Additionally, as already mentioned, juvenile material is used to initiate many embryogenic systems in woody species. Its value in the induction of somatic embryos is unknown; therefore, the plants obtained from these embryos must be evaluated in the field. The combined application of cryopreservation and somatic embryogenesis enables embryogenic or transgenic cultures to be stored while these studies last, and once these have finished, the embryogenic lines with the best characteristics could be regenerated on a large scale (Park et al., 1998). The combination of these two techniques (somatic embryogenesis and cryopreservation) makes it possible to develop high value clonal silviculture (Weng et al., 2010) as is now being applied in countries advanced in the forestry sector. It is also known that the proliferation of embryogenic cultures for long periods of time can lead to losses due to contamination, somaclonal variation, and decrease in its capacity to generate embryos, which together with high maintenance costs makes it necessary to develop protocols for their medium and long-term conservation.

To avoid cell damage during freezing and defrosting processes, the explants must be previously treated with cryoprotective solutions (vitrification) or with progressive desiccation treatments (Corredoira et al., 2017a). Desiccation involves the dehydration of plant material (in a laminar airflow cabinet, over silica gel, or by using a flow of sterile compressed air (flash drying)) and subsequent direct immersion in LN (Corredoira et al., 2017a). Vitrification is probably one of the techniques that has been applied most in plants in the past few years. Basically, it is the conversion of the water from the cells into a non-crystalline, amorphous solid, by means of the increase in its viscosity which prevents the formation and subsequent growth of ice crystals. The vitrification solutions are concentrated solutions of cryoprotective substances, such as ethylene glycol, dimethyl sulfoxide (DMSO), polyethylene glycol, and glycerol, generally combined with a high concentration of sucrose. The vitrification solution most employed is PVS2 (plant vitrification solution 2) consisting of glycerol (30% w/v), ethylene glycol (15% w/v), and DMSO (15% w/v) in liquid medium with 0.4 M sucrose (Sakai et al., 1990).

In holm oak, the cryostorage techniques have been applied for the storage of zygotic and somatic embryos with varied success. Cryopreservation of holm oak zygotic embryo axes was attempted using a desiccation protocol at different cooling rates, but no plantlets were recovered, as embryo axes of this species are sensitive to dehydration and freezing (González-Benito et al., 2002). Although cryopreservation is considered the most promising procedure for long-term conservation of intermediate and recalcitrant seeds (Ballesteros et al., 2014), research in this area is still at a very preliminary stage and a few successful studies have been performed in Fagaceae species (Corredoira et al., 2017a). In one of them, González-Benito and Pérez-Ruiz (1992) achieved a germination rate of 60% from cryostored embryonic axes of *Q. faginea*. Similarly, Corredoira et al. (2004) obtained 63% plantlet regeneration from cryostored embryonic axes of European chestnut. In both studies, desiccation treatment in the airflow of a laminar flow cabinet was applied before to the LN immersion.

**Figure 3:**
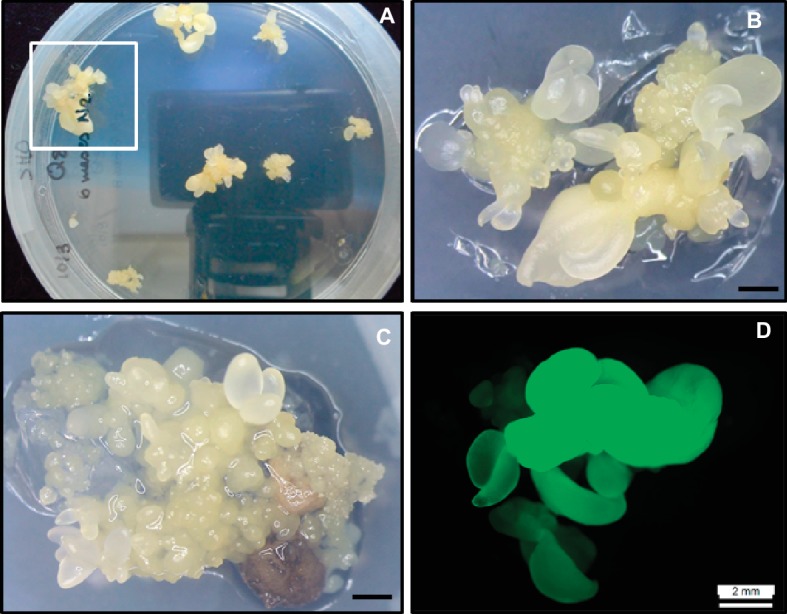
Cryopreservation and genetic transformation of embryogenic cultures of holm oak. **(A)** Somatic embryo clumps generated from cryopreserved nodular embryogenic structures following exposure to PVS2 solution for 15 min and 6 months in LN. **(B)** High magnification of A (square) to show somatic embryos generated from a cryopreserved nodular structure. Scale bar: 1 mm. **(C)** Transgenic somatic embryos and nodular embryogenic structures after transformation with EHA105pTAU strain and 10 weeks on kanamycin medium. Scale bar: 1 mm. **(D)** Somatic embryos showing green fluorescence under UV light.

On the contrary, there are currently efficient and reproducible protocols available for the cryopreservation of embryogenic cultures of oak species and other species of Fagaceae family (Vieitez et al., 2012; Corredoira et al., 2017a). In holm oak, Barra-Jiménez et al. (2015) reported a vitrification method for the cryopreservation of embryogenic lines. Globular explants of three embryogenic lines were precultured on medium with 0.3 M sucrose and then incubated for 30 min in the PVS2 solution before being submerged in LN. Embryo recovery was observed in somatic embryos stored 24 h in LN, but not in somatic embryos stored for 1 month, in which only callus formation was observed. Recently, successful embryo recovery was obtained from holm oak embryogenic lines stored in LN for 6 months (Figures 3A,B), by means of the reduction in exposure time to PVS2 from 30 to 15 min, and using isolated NSs instead of globular embryos (Corredoira et al., 2017a). The results obtained in previous works in other Fagaceae, such as chestnut (Corredoira et al., 2004), cork oak (Valladares et al., 2004), and different American oak species (Vieitez et al., 2012; Corredoira et al., 2017a), support the fact that the choice of the explant type to cryopreserve and the time of exposure to the PVS2 solution are determining factors for the recovery of the embryogenic capacity after cryopreservation.

## Genetic Transformation of Embryogenic Cultures

The combination of somatic embryogenesis with genetic transformation is a powerful tool for the improvement of forest species (Sutton, 2002). The selection of a determined characteristic in a woody species using conventional improvement techniques is slow and tedious process of crossings and selections that require several decades to achieve the desired objective. The genes that control resistance to the most important diseases affecting holm oak have not yet been identified. However, genomic tools are developing rapidly, and in the near future it should be possible to identify these genes in both this and other members of the family (chestnut, pedunculate oak and cork oak), which suffer the same or similar diseases. In this sense, the main objective of the Hardwood Genomics Project^1^ is the sequencing of the transcriptomes of chestnut, oak, and beech, with the aim, among others, of isolating the genes that regulate the resistance to diseases that affect them (Wheeler and Sederoff, 2009). Barakat et al. (2009) have compared the transcriptomes of the American (susceptible species) and Asian chestnuts (resistant species) as a response to the canker infection suffered by the American chestnut. Similarly, Serrazina et al. (2015) compared the transcriptomes of roots of the *C. sativa* (susceptible species) and *C. crenata* (resistant species) after *P. cinnamomi* infection. Both authors have identified candidate genes differentially expressed in sick and health trees associated with the plant defenses, providing new candidate genes that could favor resistance to the diseases that affect them and help in the study of the response of the trees to the pathogens. Likewise, the most important information on the oak genome, including the identification of the genes involved in the adaption of the species to specific stresses (including biotic), has been reviewed in detail (Kremer et al., 2007, 2012). These authors also show that, of the gene maps developed for chestnut and oak, it appears obvious that the molecular markers could be easily transferred from one species to another, on being very close phylogenetically. Other omic technologies, such as proteomic and metabolomic studies performed in the last years on holm oak, may also provide a better understanding of molecular mechanisms involved on oak decline (Valero-Galván et al., 2011; Sghaier-Hammami et al., 2013).

Meanwhile, since the specific genes have still not been identified, it is necessary to look for alternatives with the aim of inducing, although it may be generic, some type of fungal resistance in these forest species. An alternative is the overexpression of pathogenesis-related proteins. These proteins comprise a group of diverse proteins for which accumulation is triggered by pathogen attack, abiotic stress, hypersensitive response, and systemic acquired resistance (Van Loon, 1997; Veluthakkal and Dasgupta, 2010). Among these, genes encoding for thaumatin-like proteins have been used in order to enhance plant resistance to fungal diseases (Popowich et al., 2007). Using this strategy, Cano et al. (2017) transformed, for the first time, holm oak somatic embryos with a thaumatin-like protein of 23-KD, termed *CsTL1*, and purified from mature European chestnut cotyledons (García-Casado et al., 2000). Two to three PEMs/NSs were isolated from the Q8 holm oak embryogenic line and pre-cultured for 1 day, 1 week, or 2 weeks on SH medium without PGRs. After preculture time, explants were co-cultured for 5 days with *Agrobacterium tumefaciens* strain EHA105 harboring the pTAU binary vector. Explants were then transferred onto selective medium consisting of SH medium containing kanamycin (100 mg/L) and carbenicillin (300 mg/L). After cocultivation, explants gradually turned brown and some showed signs of necrosis. Newly emerging embryos or structures were observed in necrotic explants after 6–8 weeks of culture (Figure 3C). Only grown explants were isolated after 10 weeks of culture on selective medium. Then, these resistant somatic embryos were transferred to fresh selective medium for further 4 weeks. Following this period (14 weeks in total), surviving explants were evaluated on the basis of GFP expression (Figure 3D) to determine the transformation efficiency. The best results (2%) were obtained with explants precultured for 1 week. Transformation rates mentioned for other oak species were also relatively low. For example, in pedunculated oak, transformation rates of 1.4–9.6% were published (Mallón et al., 2014). In spite of that, a total of 11 transformed lines were obtained in holm oak, all of which were maintained by secondary embryogenesis and cryostored on LN while their resistance was evaluated. Plants were successfully produced from transformed somatic embryos stored for 2 months at 4°C.

## Concluding Remarks and Future Perspectives

Holm oak, an ecological and economically important tree species of the Mediterranean basin, is subject to possible disappearance as result of unstoppable progress of oak decline disease. Vegetative propagation of disease resistant/tolerant trees on natural populations of holm oak can be a possible solution. In the last few years, great efforts have been made in the development of vegetative propagation procedures in holm oak, especially by somatic embryogenesis. As a result of these efforts, several procedures for somatic embryogenesis induction on different explants derived from adult trees of holm oak have been described. For holm oak somatic embryogenesis induction, the use of apex explants excised from axillary shoot cultures to induce somatic embryos is highly recommended. The efficient embryogenic systems available for holm oak have led, for the first time, to the development of a genetic transformation protocol in this species. This protocol has not only enabled plants to be obtained that overexpress a thaumatin-like protein but also opened the possibility of evaluating new, more specific, genes that could confer resistance to the disease that affects them. Additionally, efficient cryopreservation procedures have been defined, which enable embryogenic lines and transgenic embryogenic lines generated to be conserved with minimal cost while regenerated plantlets are evaluated to *P. cinnamomi* tolerance/resistance in the field. A key element in the proliferation, as well as in the genetic transformation and cryopreservation, is the use of NSs as initial explant.

In the last years, knowledge about gene regulation of plant differentiation has speed out and genes involved on the induction of embryogenic competence of somatic cells have been identified in woody plant species, mainly conifers (Rupps et al., 2016; Gautier et al., 2018). But, in oaks and in spite of its ecological and economical importance, very few reports have been released concerning genetic control of SE induction (Šunderlíková et al., 2009; Valladares et al., 2013) and maturation (Šunderlíková and Wilhem, 2002; Pérez et al., 2015). More recently, DNA demethylation has been associated with SE initiation in *Q. alba* (Corredoira et al., 2017b). Proteomic changes during the embryogenic process have been studied in *Q. suber* (Gómez-Garay et al., 2013), and a multi-omics analysis has been approached in holm oak leaves (López-Hidalgo et al., 2018). All these data along with the recent release of the oak genome (Plomion et al., 2018) provide useful information that may help to improve mass clonal propagation of the recalcitrant holm oak species.

## Author Contributions

IA and MM wrote the SE induction on floral explants sections, plantlet acclimatization, concluding remarks, and future perspectives. EC and MTM prepared the figures and wrote the entire manuscript with the exception of the abovementioned sections. MS-J, VC, IA, MM, and MC improved and revised the original draft and figures. All authors read and approved the final manuscript.

### Conflict of Interest Statement

The authors declare that the research was conducted in the absence of any commercial or financial relationships that could be construed as a potential conflict of interest.

## Footnotes

^1^www.hardwoodgenomics.org
